# Elevated level of multibranched complex glycan reveals an allergic tolerance status

**DOI:** 10.1186/s12014-024-09491-8

**Published:** 2024-06-08

**Authors:** Ran Zhao, Chao Wang, Feidie Li, Zeyu Zeng, Yijing Hu, Xiaoyan Dong

**Affiliations:** 1 Department of Respiration, School of Medicine, Shanghai Children’s Hospital, Shanghai Jiao Tong University, 355 Luding Rd Shanghai, Shanghai, 200062 China; 2 Institute of Pediatric Infection, Immunity, and Critical Care Medicine, Shanghai Jiao Tong University School of Medicine, Shanghai, China; 3 NHC Key Laboratory of Medical Embryogenesis and Developmental Molecular Biology & Shanghai Key Laboratory of Embryo and Reproduction Engineering, Shanghai, China

**Keywords:** Allergen immunotherapy, Allergic patients, *N-*glycomics, Mass spectrometry, Serum

## Abstract

**Background:**

Allergen immunotherapy (AIT) is the only disease-modifying therapy that can achieve immune tolerance in patients through long-term allergen stimulation. Glycans play crucial roles in allergic disease, but no information on changes in glycosylation related to an allergic tolerance status has been reported.

**Methods:**

Fifty-seven patients with house dust mite (HDM) allergies were enrolled. Twenty-eight patients were not treated with AIT, 19 patients had just entered the AIT maintenance treatment phase, and 10 patients had been in the AIT maintenance phase for more than 1 year. Serum protein *N-*glycans were analyzed by matrix-assisted laser desorption ionization-time of flight mass spectrometry (MALDI-TOF MS), which included linkage-specific sialylation information.

**Results:**

Eighty-four *N*-glycans were identified in all three groups. Compared with the patients treated without AIT, the patients treated with AIT for a shorter time showed downregulated expression of high-mannose glycans and upregulated expression of α2,6 sialic acid. The patients treated with AIT in the maintenance phase for over 1 year, which was considered the start of immunological tolerance, showed downregulated expression of biantennary *N-*glycans and upregulated expression of multibranched and complex *N-*glycans. Nine *N-*glycans were changed between allergic and allergic-tolerant patients.

**Conclusions:**

The glycan form changed from mannose to a more complex type as treatment time increased, and multibranched complex glycans have the potential to be used as a monitoring indicator of immune tolerance. This serum *N*-glycome analysis provided important information for a deeper understanding of AIT treatment at the molecular level.

**Supplementary Information:**

The online version contains supplementary material available at 10.1186/s12014-024-09491-8.

## Background

The incidence of allergic diseases is increasing every year. Allergic rhinitis is the most common allergic disease of the respiratory system, is often accompanied by allergic asthma, and has a major impact on sleep quality and work or school and sports performance [1]. Symptomatic treatment and anti-allergy medications can only provide partial relief. The only disease-modifying therapy currently available for allergic diseases is allergen immunotherapy (AIT), a treatment regimen that achieves immune tolerance by stimulating the patient with allergens for up to 3–5 years [2]. Allergies are characterized by Immunoglobulin E (IgE)-mediated degranulation of mast cells, which in turn induces a Th2 response that promotes the production of interleukin (IL)-4, IL-5, IL-9 and IL-13 [3]. After desensitization, dendritic cells produce IL-10 [4] and IL-12 [5], the Th2 (T helper type 2) response is suppressed [6], and regulatory T- [7] and B-cell [8] responses are increased, facilitating the conversion of IgE produced by B cells into Immunoglobulin G (IgG)4 [9] and IgA [10]. This conversion leads to the suppression of IgE-mediated mast cell and basophil activation, IgE-promoted antigen presentation, and Th2 responses in the allergic response [11, 12]. The efficacy and safety of AIT treatment have been largely confirmed, and current patient assessment is mainly based on subjective evaluation [2]. Finding appropriate laboratory indicators may help to effectively monitor therapeutic responses and predict therapeutic success.

Glycosylation is the most common posttranslational modification of proteins and is catalyzed by glycosyltransferases in the endoplasmic reticulum and Golgi apparatus [13]. Glycosylation is mainly divided into *N-*glycosylation and *O-*glycosylation [14] and is very sensitive to changes in the biochemical environment and physiopathology and can be used as a marker for cancer [15, 16], Alzheimer's disease [17], rheumatoid arthritis [18, 19], and many other diseases. Proteins have been widely used as basic disease markers, but there are limitations such as the inability to differentiate between disease states, typing, drug-sensitive phenotypes and so on, which can be corrected and refined when combined with glycosylation [20–22].

Glycosylation of immune globulins plays critical roles in allergic diseases. The N394-linked oligo-mannose glycosylation of IgE is a key factor in its binding to FcεRI, resulting in a Th2 response [23]. When this glycosylation is altered, the binding capacity of IgE is altered [23, 24]. Sialic acid glycan modification on IgE has also been reported to be involved in the induction of allergic reactions, and the absence of sialic acid modification on IgE can effectively reduce peanut allergic symptoms [25]. Various glycan-binding proteins can elicit different immune responses depending on the type of glycan they encounter, thereby activating the immune response [26]. For example, sialic acid causes the accumulation of white blood cells at the site of inflammation [27], and *O-*GlcNAcylation can affect the polarization of macrophages [28]. Therefore, the specific glycosylation of allergens can be more effectively targeted by immune cells, which represent attractive targets for AIT and thus achieve immune tolerance at low doses [29]. However, the mechanism of the immune response in vivo after desensitization is still unclear. There are many inflammatory proteins and antibodies in the blood, and the glycosylation changes in serum protein itself before and after desensitization are not clear. After desensitization, serum glycomic changes at different time points can provide more sensitive and stable monitoring markers for therapeutic effects and status changes from allergy to allergic tolerance. Assessment of these changes can also provide omics data to help reveal the mechanism of glycosylation involved in allergic reactions.

House dust mites (HDMs) are one of the most common allergens. AIT treatment can be divided into two major phases: a dose escalation phase, in which the dose of HDM allergen injected in the arm is raised weekly, and a maintenance phase, after the dose escalation phase, in which the highest dose of HDM allergen is maintained and injected in the arm every 4–6 weeks. We analyzed the serum *N-*glycomics of 57 HDM allergic patients, 28 of whom did not participate in AIT treatment and 29 of whom were treated with AIT (subcutaneous immunotherapy, SCIT). Nineteen patients enrolled in this study had just entered the maintenance phase of AIT treatment (time 1 after treatment), and the other ten patients had been in the maintenance phase for more than 1 year (time 2 after treatment), which was considered the start of immunological tolerance [9]. For glycomic analysis, we chose a simple, high-throughput, and less serum-intensive method, as previously reported [30], that allows the discrimination of α2,3 and α2,6 sialic acids by esterification. Eighty-four *N-*glycans were identified in the three groups, and the glycosylation differences could represent serum markers of immune tolerance achieved by AIT treatment.

## Methods

### Study population and sample collection

We enrolled 57 patients with HDM allergy, 28 of whom were not receiving AIT. The SCIT of dust mite allergens was carried out using dust mite allergens prepared by the Danish ALK company and the methods recommended in the instructions. The treatment course was divided into a dose-accumulation stage and a maintenance stage; 19 patients had received AIT at the highest dose for the first time and had just entered the maintenance phase, and 10 were in the maintenance phase, and the highest dose was injected for more than 1 year. After their own subjective evaluation (visual analogue scale (VAS) scores decreased means the symptoms improved [31]), the therapeutic effect of all patients improved. We collected peripheral blood before the patients were injected with allergens. The specific IgE (sIgE) levels of derp1, derp2 were calculated by double antibody sandwich enzyme-linked immunosorbent assay (ELISA, Hangzhou Zheda Dixun Biological Gene Engineering Co., Ltd). All patients (or their parents) signed informed consent forms, and the study was approved by the ethics commission of the Shanghai Children's Hospital ethics committee (2020RY140-E01).

### Serum N-glycan preparation

Blood was collected from patients and allowed to settle at room temperature for 30 min, followed by centrifugation at 3000 rpm for 15 min. Then, the supernatant was collected and stored at −80 °C. *N-*glycans were enzymatically released from patient serum as previously reported [32]. Briefly, five microliters from each sample was denatured by adding 10 μL of 2% sodium dodecyl sulfate (SDS) (Merck, Darmstadt, Germany) and incubated for 10 min at 60 °C. Subsequently, the denatured samples were added to 10 μL of 2.5 × phosphate buffered saline (PBS) containing 2% Nonidet P-40 (New England Biolabs) and 1 mU Peptide *N-*Glycosidase F (PNGase F) (New England Biolabs) and incubated for 16 h at 37 °C.

### Sialic acid derivatization and purification of N-glycans

Two microliters of released *N-*glycans was added to 20 μL of derivatization reagent (250 mM 1-(3-dimethylaminopropyl)-3-ethylcarbodiimide (EDC) and 250 mM 1-hydroxybenzotriazole (HOBt) dissolved in ethanol) and incubated at 37 °C for 60 min [30]. Using a combination of carboxylic acid activators in ethanol achieved near-complete ethyl esterification of α2,6-linked sialic acids and lactonization of α2,3-linked variants, in short time using mild conditions[30]. Then, 22 μL of acetonitrile (ACN) was added and incubated at −20 °C for 15 min, and the proteins were removed. Thereafter, hydrophilic interaction liquid chromatography solid-phase extraction (HILIC-SPE) tips made of cotton were used to purify glycans as reported in previous studies [33].

### MALDI-TOF–MS analysis and data processing

The measurement of the derivatized glycans was performed on a Bruker ultrafleXtreme laser MALDI-TOF mass spectrometer equipped with Smartbeam-II in reflected positive ion mode and commanded by the proprietary software Flexcontrol 3.4 (Bruker Daltonics). One microliter of each sample was placed on an MTP 384 target plate polished steel BC (Bruker Daltonics), and 1 μL of substrate (newly prepared 5 mg/mL super- 2,5-Dihydroxybenzoic acid (DHB), dissolved in 1 mM NaOH in 50% ACN) was added and dried at room temperature. Each sample spot was analyzed in 3 duplicates. The *m/z* range was monitored to range from 1000 to 4000 with 10,000 laser shots using a complete-sample random walk of 100 shots per spot at a frequency of 1000 Hz, and the laser voltage was set at 80 V. The mass spectrograms were analyzed using flexAnalysis (Bruker Daltonics) and further imported into commercial BioPharma Compass software (Bruker Daltonics) to extract the peak signal intensity of each *N-*glycan spectrum. Eighty-four glycans were identified. The relative peak intensities were calculated as previously described [22], and the relative intensity of each type of *N-*glycan was calculated by dividing the intensity of a given type of *N-*glycan by the total 84 *N-*glycan intensity(raw data and transfer formulas are shown in the supplemental material 1). Then, the total intensity was normalized to 100, and 9 derived glycosylation traits were further calculated (calculation formulas are shown in the supplemental material 2).

### Statistical analysis

Before the statistical analyses, the relative intensities of 84 glycans were log10-transformed to obtain values closer to a normal distribution. The differences between glycans were analyzed using an unpaired Student’s t test with SPSS software (version 16.0). The two AIT-treated groups were compared with the untreated AIT group (p < 0.05 was considered statistically significant). Multiple testing correction was further conducted to adjust the significance threshold more rigorously for each *N-*glycan (p < 0.05/84, 84 is the number of *N-*glycan traits). In the two AIT-treated groups, the VAS score differences before and after AIT treatment of each patient were analyzed using the paired samples Wilcoxon signed rank test. All statistical analyses were conducted in SPSS software version 26.

## Results

### Patient characteristics

**Fig. 1:**
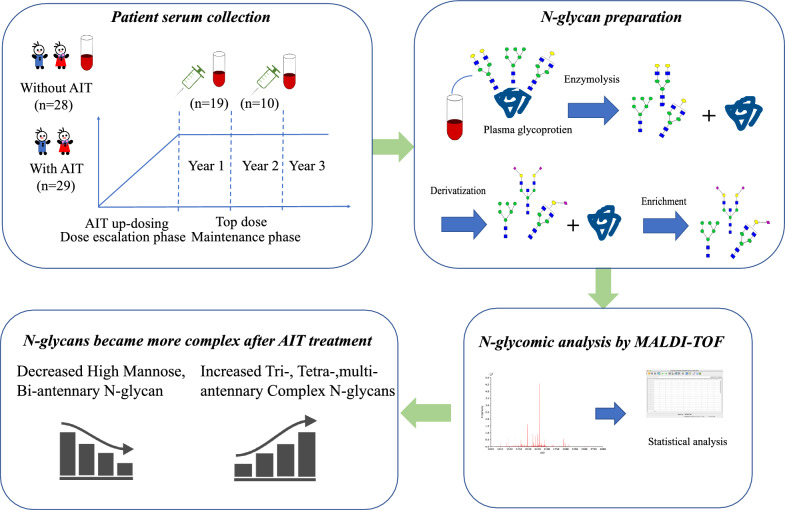
The outline diagram of the whole process to investigate the different *N-*glycan expression levels between patients treated without AIT and those treated with AIT

There were 57 patients with dust mite allergy (grade 1 or higher), including 28 patients (13 males, 15 females) without treatment, with a mean age of 7.5 years (range 5–13), 11 patients diagnosed with rhinitis, 3 patients with asthma and 14 patients with rhinitis mixed with other allergic disease (asthma/conjunctivitis/psoriasis/cough variant asthma). Nineteen patients had just entered the maintenance period (10 males, 9 females), with a mean age of 8.5 years (range 5–13) and a median treatment time of 23 weeks (16–36); 7 of these patients had rhinitis, and 12 patients had rhinitis mixed with other allergic disease. Finally, 10 patients (6 males, 4 females) had been treated with AIT at the highest dose for more than 1 year, with a mean age of 11.2 years (range 9–15) and a median treatment time of 95 weeks (84–142); 2 of these patients had rhinitis, 3 had asthma and 5 had rhinitis mixed with other allergic disease. The sIgE degrees of Derp1 and Derp2 are shown in Table 1. These patients were enrolled to investigate the differences in *N-*glycan expression between patients treated without AIT or with AIT to achieve allergic tolerance. The whole process is shown in the outline diagram (Fig. 1).

**Table 1:** Clinical characteristics of allergy patients under study

	Without treatment	Time 1 after treatment	Time 2 after treatment
Number (male, female)	28 (13,15)	19 (10,9)	10 (6,4)
Age (years)	7.5 (5–13)	8.5 (6–13)	11.2 (9–15)
Weeks of treatment	0	23 (16–36)	95 (84–142)
Diagnosis (Patient number)			
Rhinitis alone	11	7	2
Asthma alone	3	0	3
Mixed allergic diseases	14	12	5
SIgE degree of Derp1,Derp2 (IU/mL)			
Degree 1 (0.35–0.69)	1	0	0
Degree 2 (0.7–3.49)	4	2	2
Degree 3 (3.5–17.49)	9	3	4
Degree 4 (17.5–49.9)	7	8	3
Degree 5 (50–100)	6	4	1
Degree 6 (> 100)	1	2	0

Mixed allergic diseases: Rhinitis mixed with asthma/conjunctivitis/psoriasis/cough variant asthma. Derp1, Derp2: Dermatophagoides pteronyssinus, main allergen components of house dust mites, *sIgE* specific IgE

### Alteration of serum N-glycans between each AIT-treated group and the untreated group of patients

**Fig. 2:**
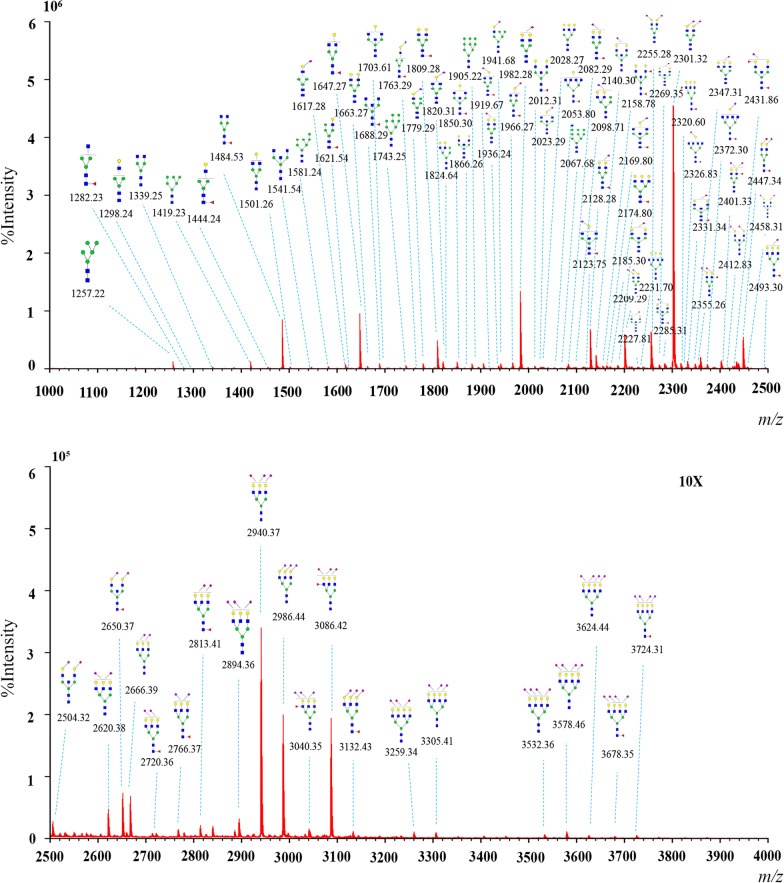
Typical MALDI-TOF–MS spectra of allergic patients. The *m/z* range of the upper spectra is from 1000–2500. The *m/z* range of the lower spectra was 2500–4000, and the peak intensities were amplified tenfold to clearly show the peak. *N*-acetylglucosamine (GlcNAc): blue square; mannose (Man): green circle; galactose (Gal): yellow circle; sialic acid (NeuAc): purple lozenge; fucose (Fuc): red triangle. All cartoons described the possible structure matching the composition (other structure possibilities are not excluded)

**Fig. 3:**
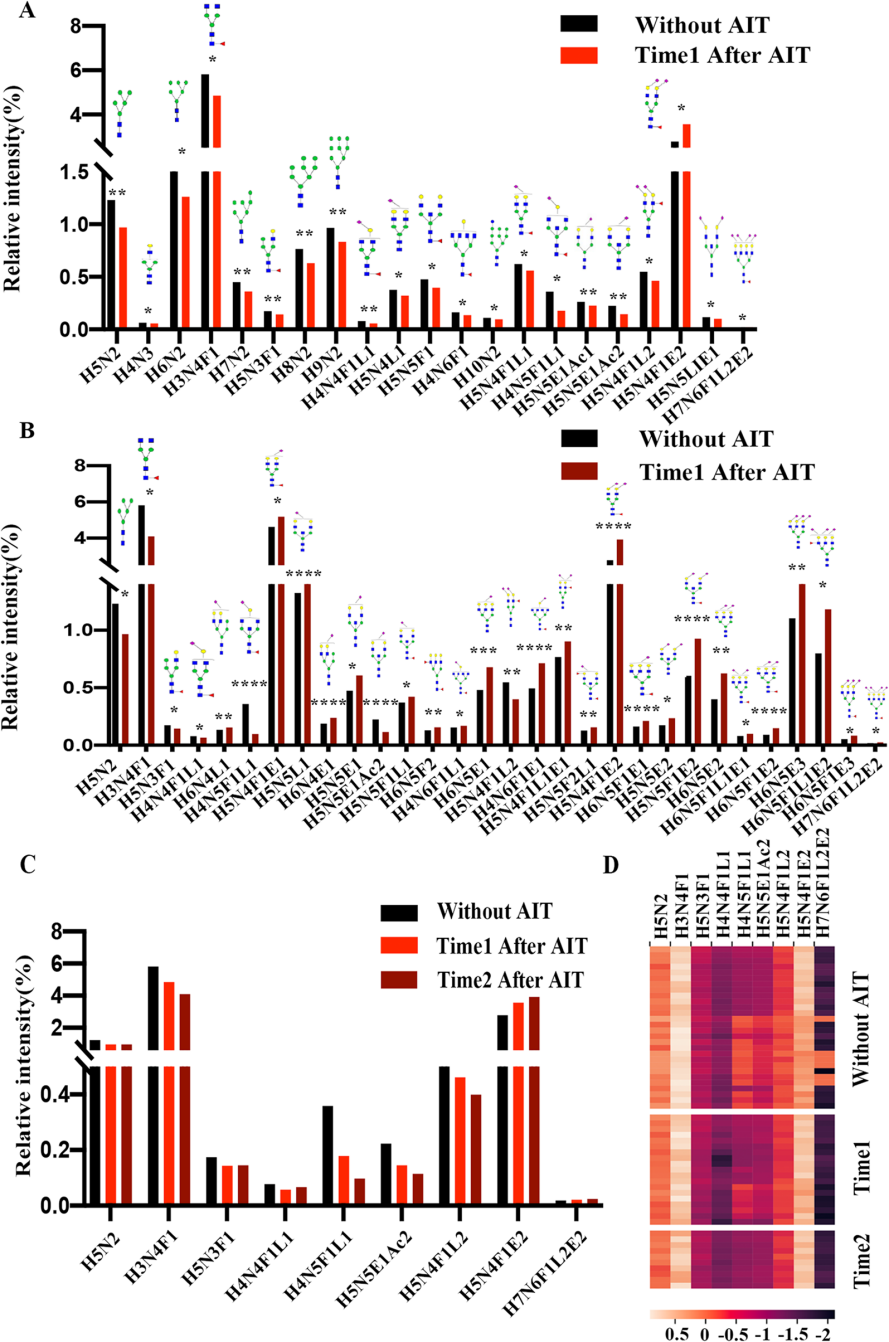
Comparison of statistically significant relative intensities between the allergic patients treated without AIT and the allergic patients treated with AIT who had just entered the maintenance phase (**a**) and the allergic patients treated with AIT who had been in the maintenance phase over 1 year (**b**). The 84 most intense peaks were compared, and only statistically significant peaks are shown (*p < 0.01; **p < 0.05; *** p < 0.001; multiple testing correction was conducted: **** p < 0.05/84). The levels of nine *N-*glycans were changed constantly at two time points after AIT. The relative intensities of the nine *N-*glycans (**c**); Heatmap made by logging the relative intensities of these nine *N-*glycan chains in 57 patients with a base of 10 (**d**). Without AIT: patients were not treated with AIT; Time 1 after treatment: patients were treated with AIT and had just entered the maintenance phase; Time 2 after treatment: patients were treated with AIT and had been in the maintenance phase for 1 year. All cartoons described the possible structure matching the composition (other structure possibilities are not excluded)

Serum *N-*glycans were analyzed in allergic patients without and with AIT treatment, and 84 *N-*glycans were identified in all groups (Fig. 2 and Table 2). The proposed glycan structures were reported based on previous articles [34, 35]. The levels of 21 *N-*glycans were significantly changed between the 19 patients who had just entered the maintenance phase and the 28 patients without treatment: H5N2, H4N3, H6N2, H3N4F1, H7N2, H5N3F1, H8N2, H9N2, H4N4F1L1, H5N4L1, H5N5F1, H4N6F1, H10N2, H5N4F1L1, H4N5F1L1, H5N5E1Ac1, H5N5E1Ac2, H5N4F1L2 and H5N5LIE1 (*m/z* 1257.22, 1298.24, 1419.23, 1484.53, 1581.24, 1621.54, 1743.25, 1905.22, 1919.67, 1936.24, 2012.31, 2053.80, 2067.68, 2082.29, 2123.75, 2227.81, 2269.35, 2355.26 and 2458.31) were significantly decreased, and H5N4F1E2 and H7N6F1L2E2 (2447.34 and 3724.31) were significantly increased (p < 0.05) (Fig. 3a). There were 30 significantly changed *N-*glycans between the 10 patients who were treated in the maintenance phase for more than 1 year and considered allergic tolerant and the patients without treatment. The levels of H6N4L1, H5N4F1E1, H5N5L1, H6N4E1, H5N5E1, H5N5F1L1, H6N5F2, H4N6F1L1, H6N5E1, H4N6F1E1, H5N4F1L1E1, H5N5F2L1, H5N4F1E2, H6N5F1E1, H5N5E2, H5N5F1E2, H6N5E2, H6N5F1L1E1, H6N5F1E2, H6N5E3, H6N5F1L1E2, H6N5F1E3 and H7N6F1L2E2 (*m/z* 2098.71, 2128.28, 2140.30, 2144.75, 2185.30, 2285.31, 2320.60, 2326.83, 2347.31, 2372.30, 2401.33, 2431.86, 2447.34, 2493.30, 2504.32, 2650.37, 2666.39, 2766.37, 2813.41, 2986.44, 3086.42, 3132.43 and 3724.31) were significantly elevated, and the levels of H5N2, H3N4F1, H5N3F1, H4N4F1L1, H4N5F1L1, H5N5E1Ac2 and H5N4F1L2 (*m/z* 1257.22, 1484.53, 1621.54, 1919.67, 2123.75, 2269.35 and 2355.26) were significantly reduced (Fig. 3b).

**Table 2:** Relative intensities of proposed *N-*glycan structures in patients with or without AIT

	*m/z*	Composition	Proposed structure	Without AIT treatment	Time 1 after treatment	Time 2 after treatment
1	1257.22	H5N2		1.231	0.969 **	0.965 *
2	1282.23	H3N3F1		0.067	0.062	0.062
3	1298.24	H4N3		0.063	0.055 *	0.057
4	1339.25	H3N4		0.080	0.088	0.066
5	1419.23	H6N2		1.535	1.260 *	1.321
6	1444.24	H4N3F1		0.075	0.067	0.072
7	1484.53	H3N4F1		5.813	4.849 *	4.089 *
8	1501.26	H4N4		0.243	0.247	0.246
9	1541.54	H3N5		0.086	0.078	0.069
10	1581.24	H7N2		0.448	0.360 **	0.439
11	1617.28	H4N3E1		0.493	0.494	0.552
12	1621.54	H5N3F1		0.174	0.143 **	0.145 *
13	1647.27	H4N4F1		7.933	7.051	7.064
14	1663.27	H5N4		0.415	0.365	0.428
15	1688.29	H3N5F1		0.765	0.645	0.639
16	1703.61	H4N5		0.117	0.109	0.116
17	1743.25	H8N2		0.764	0.630 **	0.785
18	1763.29	H4N3F1E1		0.147	0.147	0.157
19	1779.29	H5N3E1		0.539	0.514	0.561
20	1809.28	H5N4F1		4.597	4.212	4.873
21	1820.31	H4N4E1		0.637	0.611	0.686
22	1824.64	H6N4		0.088	0.083	0.096
23	1850.30	H4N5F1		1.185	1.036	1.328
24	1866.26	H5N5		0.115	0.103	0.120
25	1905.22	H9N2		0.964	0.832 **	1.063
26	1919.67	H4N4F1L1		0.078	0.057 **	0.066 *
27	1936.24	H5N4L1		0.377	0.321 *	0.332
28	1941.68	H6N3E1		0.521	0.498	0.556
29	1966.27	H4N4F1E1		0.610	0.614	0.687
30	1982.28	H5N4E1		9.270	9.174	9.871
31	2012.31	H5N5F1		0.475	0.396 *	0.520
32	2023.29	H4N5E1		0.206	0.205	0.225
33	2028.27	H6N5		0.139	0.130	0.154
34	2053.80	H4N6F1		0.162	0.135 *	0.138
35	2067.68	H10N2		0.109	0.096 *	0.113
36	2082.29	H5N4F1L1		0.621	0.559 *	0.565
37	2098.71	H6N4L1		0.134	0.133	0.154 **
38	2123.00	H4N5F1L1		0.358	0.179 *	0.097 ****
39	2128.28	H5N4F1E1		4.619	4.869	5.183 *
40	2140.30	H5N5L1		1.324	1.441	1.696 ****
41	2144.75	H6N4E1		0.188	0.189	0.238 ****
42	2158.78	H5N5F2		0.102	0.094	0.103
43	2169.80	H4N5F1E1		0.270	0.259	0.275
44	2174.80	H6N5F1		0.081	0.069	0.087
45	2185.30	H5N5E1		0.474	0.473	0.606 *
46	2209.29	H5N4L2		0.255	0.233	0.236
47	2227.81	H5N5E1Ac1		0.261	0.227 **	0.246
48	2231.70	H6N6		0.108	0.095	0.111
49	2255.28	H5N4L1E1		4.704	4.771	4.598
50	2269.35	H5N5E1Ac2		0.223	0.146 **	0.114 ****
51	2285.31	H5N5F1L1		0.371	0.368	0.421 *
52	2301.32	H5N4E2		30.419	33.116	27.783
53	2320.60	H6N5F2		0.129	0.137	0.156 **
54	2326.83	H4N6F1L1		0.153	0.155	0.168 *
55	2331.34	H5N5F1E1		1.750	1.679	1.989
56	2347.31	H6N5E1		0.480	0.480	0.678 ***
57	2355.26	H5N4F1L2		0.547	0.462 *	0.399 **
58	2372.30	H4N6F1E1		0.495	0.606	0.713 ****
59	2401.33	H5N4F1L1E1		0.766	0.851	0.902 **
60	2412.83	H5N5L2		0.058	0.038	0.062
61	2431.86	H5N5F2L1		0.127	0.124	0.157 **
62	2447.34	H5N4F1E2		2.778	3.560 *	3.925 ****
63	2458.31	H5N5LIE1		0.116	0.100 *	0.117
64	2493.30	H6N5F1E1		0.162	0.162	0.211 ****
65	2504.32	H5N5E2		0.173	0.174	0.234 *
66	2620.38	H6N5L1E1		0.395	0.366	0.447
67	2650.37	H5N5F1E2		0.603	0.747	0.926 ****
68	2666.39	H6N5E2		0.400	0.426	0.625 **
69	2720.36	H6N5F1L2		0.062	0.059	0.059
70	2766.37	H6N5F1L1E1		0.081	0.083	0.100 *
71	2813.41	H6N5F1E2		0.090	0.101	0.147 ****
72	2894.36	H6N5L2E1		0.313	0.289	0.293
73	2940.37	H6N5L1E2		2.874	2.871	3.099
74	2986.44	H6N5E3		1.103	1.230	1.664 **
75	3040.35	H6N5F1L2E1		0.087	0.089	0.100
76	3086.42	H6N5F1L1E2		0.798	0.923	1.181 *
77	3132.43	H6N5F1E3		0.053	0.060	0.083 *
78	3259.34	H7N6L2E1		0.092	0.088	0.092
79	3305.41	H7N6L1E2		0.068	0.072	0.088
80	3532.36	H7N6L3E1		0.064	0.059	0.051
81	3578.43	H7N6L2E2		0.084	0.086	0.084
82	3624.44	H7N6L1E3		0.027	0.030	0.034
83	3678.35	H7N6F1L3E1		0.017	0.017	0.017
84	3724.31	H7N6F1L2E2		0.018	0.021 *	0.024 *

Without AIT treatment: patients were not treated with AIT; Time 1 after treatment: patients were treated with AIT and had just entered the maintenance phase; Time 2 after treatment: patients were treated with AIT and had been in the maintenance phase for 1 year. The *p* value was considered significant if it was below 0.05. *p < 0.05; **p < 0.01; ***p < 0.001, the multiple testing correction was further performed to adjust the significance threshold of each *N-*glycan (****: p < 0.05/84). Structure abbreviations: H, hexose; N, HexNAc; F, fucose; E, ethyl esterified *N-*acetylneuraminic acid (α2,6-linked); L, lactonized *N-*acetylneuraminic acid (α2,3-linked); Ac, O-Acetylation of *N-*glycolylneuraminic acid; symbols for proposed structures: GlcNAc, , Man, , Glc, , Fuc, , Gal, , Sialic acid,.All cartoons described the possible structure matching the composition (other structure possibilities are not excluded, for example, both core or branch fucosylation are possible)

Compared to those patients without treatment, the levels of nine *N-*glycans in the AIT-treated patients changed consistently with increasing treatment time (Fig. 3c). The levels of H5N2, H3N4F1, H5N3F1, H4N4F1L1, H4N5F1L1, H5N5E1Ac2 and H5N4F1L2 (*m/z* 1257.22, 1484.53, 1621.54, 1919.67, 2123.75, 2269.35 and 2355.26) were significantly decreased after AIT treatment, and the levels of H5N4F1E2 and H7N6F1L2E2 (*m/z* 2447.34 and 3724.31) were significantly increased. The expression of these 9 glycans in all patients is shown in a heatmap (log10 of the relative intensity). These consistent changes indicated that these glycans might play important roles during AIT treatment (Fig. 3d).

### Levels of nine glycans were changed between allergic and allergic-tolerant patients

The groups at time 1 and time 2 after treatment were evaluated for VAS scores before and after AIT treatment. After AIT treatment, the VAS score at time 1 after treatment was significantly decreased (z = −3.634, p < 0.0001), and the mean VAS score of this group changed from 4.63 (25%–75%, 2–7) to 1.32 (25%–75%, 1–2) before and after AIT treatment. The VAS score at time 2 after treatment was also significantly decreased (z = −2.388, p < 0.05), and the mean VAS score of this group changed from 2.5 (25%–75%, 1.75–4) to 0.7 (25%–75%, 0–2) before and after AIT treatment. Both groups were considered to improve after treatment. According to previous research, inflammation indicators started to change after 6 months and consistently indicated tolerance after treatment for at least 1 year [9]. Therefore, we defined the patients in the time 2 treatment group as allergic-tolerant patients who were treated with AIT for more than 80 weeks. Multiple testing correction was conducted to recognize more significant differences in glycans. After correction, there were no significantly changed *N-*glycans between time 1 after treatment and the untreated group, and there were 9 significantly changed *N-*glycans between time 2 after treatment and the untreated group. The levels of H4N5F1L1and H5N5E1Ac2 (*m/z* 2123.75 and 2269.35) were significantly decreased in patients with allergic tolerance, and the levels of H5N5L1, H6N4E1, H4N6F1E1, H5N4F1E2, H6N5F1E1, H5N5F1E2, H6N5F1E2 (*m/z* 2140.30, 2144.75, 2372.30, 2447.34, 2493.30, 2650.37, and 2813.41) were significantly increased (Fig. 3b, ****: p < 0.05/84).

### Serum-derived glycans became more complex after AIT treatment

**Fig. 4:**
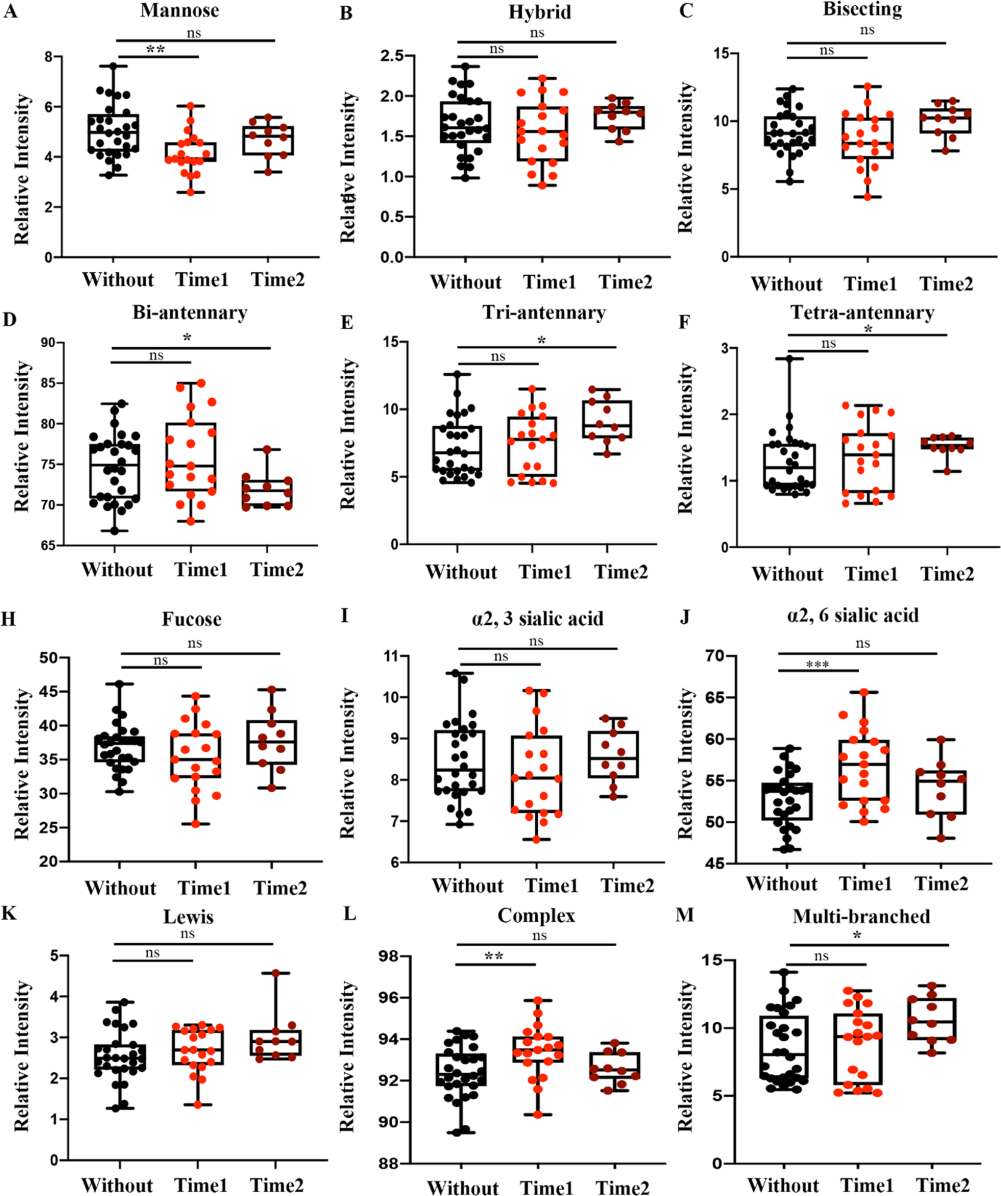
Relative abundance of different derived glycosylation traits in the three groups. Box and whisker plots of high-mannose glycans (**a**), hybrid glycans (**b**), bisecting glycans (**c**), biantennary glycans (**d**), triantennary glycans (**e**), tetra-antennary glycans (**f**), fucosylation glycans (**g**), α2,3 sialic acid glycans (**h**), α2,6 sialic acid glycans (**i**), Lewis glycans (**k**), complex glycans (**l**), and multibranched glycans (**m**). The difference between traits was compared using an unpaired Student's *t* test. The *p* value was considered significant if it was below 0.05. *p < 0.05; **p < 0.01; ***p < 0.001. “ns.” indicates no significant difference, *p* > 0.05. Without: patients were not treated with AIT; Time 1: patients were treated with AIT and had just entered the maintenance phase; Time 2: patients were treated with AIT and had been in the maintenance phase for 1 year

After classifying the *N-*glycans, the levels of 2 types of derived glycans were significantly changed in the serum of patients who had just entered the maintenance phase. The level of high mannose was significantly decreased, and the level of a2,6 sialic acid glycan was significantly elevated (Fig. 4A–J). Additionally, the levels of four types of derived *N-*glycans were significantly changed in the serum of patients in the maintenance phase of treatment for more than one year. The level of biantennary glycans was significantly reduced (Fig. 4D), and the levels of triantennary, tetra-antennary and multibranched glycans were significantly elevated (Fig. 4E–M). As the duration of treatment increased, the levels of high mannose and biantennary glycans decreased, and the levels of triantennary, tetra-antennary and multibranched complex glycans as well as a2,6 sialic acid increased, indicating a trend of simple to complex glycan forms in serum after AIT treatment (Fig. 4, supplemental material 2).

## Discussion

Many secreted proteins and immunoglobulins in the serum play activating and amplifying roles in the immune response. Glycosylation changes might be the switch involved in the conversion of the allergic response to an inhibitory state [26]. For example, IgE is absolutely needed for allergies, but IgE concentrations do not reproducibly correlate with allergic disease [25]. It was reported that glycosylation of IgE is a key factor in its allergic response [23, 25]. As another example, in T cells, T-cell antigen receptor (TCR) activation can regulate the expression of multibranched glycosyltransferases in a potential feedback loop [36], increasing the expression levels of multibranched glycans with Gal-GlcNAc repeats on TCR, which shows high affinity for galectins. Then, galectin-TCR lattice formation on the T-cell surface restricts TCR clustering to suppress T-cell activation [37]. It is likely that protein glycosylation is the key modification used to balance the immune response.

Serum glycomic profiles at two time points after AIT treatment revealed the alteration of multiple types of derived glycosylation, with glycosylation structures changing from simple to complex. The high mannose glycan level was reduced after desensitization. Mannose-type glycans are always modified on different allergens that can activate allergic responses. Establishment of the N394-linked oligo-mannose glycan of IgE is needed for appropriate IgE folding and FcεRI binding to initiate immune functions [23]. The reduction in mannose glycosylation is likely to reduce anaphylaxis, and the reduction in this serum protein glycan structure is probably related to immune tolerance. Moreover, we found that multibranched glycans were also altered after AIT, with increased expression levels of triantennary, tetra-antennary and multibranched glycans with increasing time of AIT. Multibranched glycans are mostly synthesized by *N-*acetylglucosaminyltransferase (GnT)-V synthesized by the alpha-1,6-mannosylglycoprotein 6-beta-*N-*acetylglucosaminyltransferase (mgat5) gene[38]. This structure has been reported to play a crucial role in suppressing allergic reactions. For example, it can inhibit the activation of T-cell receptor signaling. In addition, mgat5-/- mice display worse outcomes (enhanced sensitivity) in models of autoimmunity [37]. As a whole, increased multibranched glycans were indicated to be a glycan marker for the formation of immune tolerance.

The sialic acid-type glycans also changed after AIT treatment, with α2,6 sialic acids, which are mainly synthesized by ST6Gal1 and present a suppressive function in many immune reactions, showing a trend of upregulated expression after desensitization [39]. α2,6 Sialic acids on IgG Fc-localized glycans have been established to induce anti-inflammatory functions [40–42], while recognition of α2,6-sialic acids by the sialic acid-binding Ig-like lectin (Siglec) molecule on B cells selectively inhibits B-cell receptor signaling [43, 44]. This finding indicates that a long period of repeated stimulation by allergens results in an increased α2,6 sialic acid expression level in the serum proteins of patients and indicates that the immune response is gradually tolerated.

AIT is currently the only disease-modifying treatment, but the entire treatment period can take 3–5 years and may cause local and systemic adverse reactions [2]. Some patients may not improve, and others may overreact during the treatment process, and treatment may be discontinued. Whether the treatment is effective is mainly determined through the patient's subjective evaluation. There are no reliable laboratory indicators as stable markers that can be used to monitor whether the treatment has reached a tolerated state and to determine whether AIT treatment is effective. Glycosylation might be a stable indicator marker for evaluating the efficacy of this treatment. Moreover, the mechanism of immune reactions in patients after desensitization therapy is unclear, and glycosylation is likely to be an important switch involved in the regulation of balanced immunity. The number of patients in this study was limited, and more reliable evidence would be obtained if a sample of a larger population could be analyzed. We dynamically analyzed glycosylation changes in patient serum and evaluated the treatment effect of AIT from the new perspective of serum glycosylation, which may provide novel insights for the treatment of allergic patients.

### Supplementary Information

Supplementary material 1.

Supplementary material 2.

## Data Availability

Data are available within the article. All data generated or analyzed during this study are included in this published article.

## Abbreviations

AIT: Allergen immunotherapy
HDM: House dust mite
MALDI-TOF MS: Matrix-assisted laser desorption ionization-time of flight mass spectrometry
IgE: Immunoglobulin E
IL: Interleukin
SCIT: Subcutaneous immunotherapy
VAS: Visual analogue scale
SDS: Sodium dodecyl sulfate
PBS: Phosphate buffered saline
PNGase F: Peptide *N-*Glycosidase F
HILIC-SPE: Hydrophilic interaction liquid chromatography solid-phase extraction
TCR: T-cell antigen receptor
